# SOX combined with apatinib and camrelizumab in the treatment of resectable locally advanced gastric cancer: a case report

**DOI:** 10.3389/fimmu.2024.1410284

**Published:** 2024-07-12

**Authors:** JiKe Hu, Xuemei Li, Yunpeng Wang, Bo Xu, Puyi He, Zhuanfang Wang, Lijuan He, Hao Chen

**Affiliations:** ^1^ The Second Hospital and Clinical Medical School, Lanzhou University, Lanzhou, China; ^2^ Gansu Provincial Key Laboratory of Environmental Oncology, The Second Hospital and Clinical Medical School, Lanzhou, China; ^3^ Department of Surgical Oncology, The Second Hospital of Lanzhou University, Lanzhou, China

**Keywords:** SOX, apatinib mesylate, camrelizumab, locally advanced gastric cancer, case report

## Abstract

Gastric cancer is highly prevalent in China, yet early diagnosis and overall survival rates are low. The primary treatment strategy is comprehensive therapy centered on surgery. Studies indicate that neoadjuvant chemotherapy can enhance radical resection rates and extend survival in locally advanced gastric cancer. Combining VEGFR inhibitors with chemotherapy improves efficacy in digestive system tumors, while PD-1/PD-L1 inhibitors combined with anti-angiogenesis agents or chemotherapy show synergistic effects. This report presents a case of gastric adenocarcinoma (cT3N1M0) treated with SOX, apatinib mesylate, and camrelizumab as neoadjuvant therapy, followed by D2 distal gastrectomy and postoperative adjuvant therapy with the same regimen. The patient completed all treatment cycles successfully. Post-neoadjuvant therapy, only focal residual cancer cells were found in the lamina propria (pT1a). During postoperative adjuvant therapy follow-up, gastroscopic biopsy indicated a pathological complete response with no recurrence or metastasis. The patient primarily experienced dyspepsia, oropharyngeal pain, capillary proliferation, mild bone marrow suppression, nausea, and vomiting as side effects. Therefore, SOX combined with apatinib mesylate and camrelizumab shows promise for treating resectable locally advanced gastric cancer.

## Introduction

Gastric cancer is the third most common malignancy in China, accounting for 44.1% of the global incidence (1). However, the early diagnosis rate is only around 20%, and the overall 5-year survival rate is less than 50%. Most patients are diagnosed with advanced gastric cancer, characterized by invasion beyond the submucosa into the muscularis propria or beyond, with or without lymph node metastasis but without distant metastasis. Neoadjuvant chemotherapy can reduce tumor size and TNM stage, thereby increasing the likelihood of achieving R0 resection (2). Currently, for locally advanced gastric cancer (T3/4, N+), a dual regimen of platinum and fluorouracil or a triple regimen including a taxane is recommended. A recent study (3) indicates that the three-year disease-free survival rate was significantly higher in patients who received perioperative chemotherapy with SOX compared to the adjuvant chemotherapy group (61.7% vs. 53.8%, log-rank p = 0.019). Furthermore, the R0 resection rate was higher in the perioperative chemotherapy group (94.9% vs. 83.7%, p < 0.0001). A study (4) of neoadjuvant therapy with apatinib combined with oxaliplatin and capecitabine showed an objective response rate (ORR) of 78.1%, an R0 resection rate of 96.9%, and a pathological complete response (pCR) rate of 6.3%. The median event-free survival was 42.6 months. Neoadjuvant therapy with camrelizumab in combination with SOX (5) achieved encouraging results, including a pathological complete response (pCR) rate of 10.3%, an R0 resection rate of 96.6%, a major pathological response (MPR) rate of 69.0%, an objective response rate (ORR) of 93.1%, and a disease control rate (DCR) of 100%. In 2024, a phase 1 study (6) of first-line therapy with camrelizumab plus apatinib and chemotherapy for advanced gastric cancer showed an objective response rate of 76.5% and a median progression-free survival of 8.4 months. The median overall survival (OS) was not yet mature. The potential benefits of adding molecular targeted drugs or immune checkpoint inhibitors to neoadjuvant and postoperative adjuvant chemotherapy are still under investigation. This case was treated with a combination of SOX (S-1 plus oxaliplatin), apatinib mesylate, and camrelizumab to assess the perioperative treatment efficacy in resectable locally advanced gastric cancer.

## Clinical information

**Figure 1:**
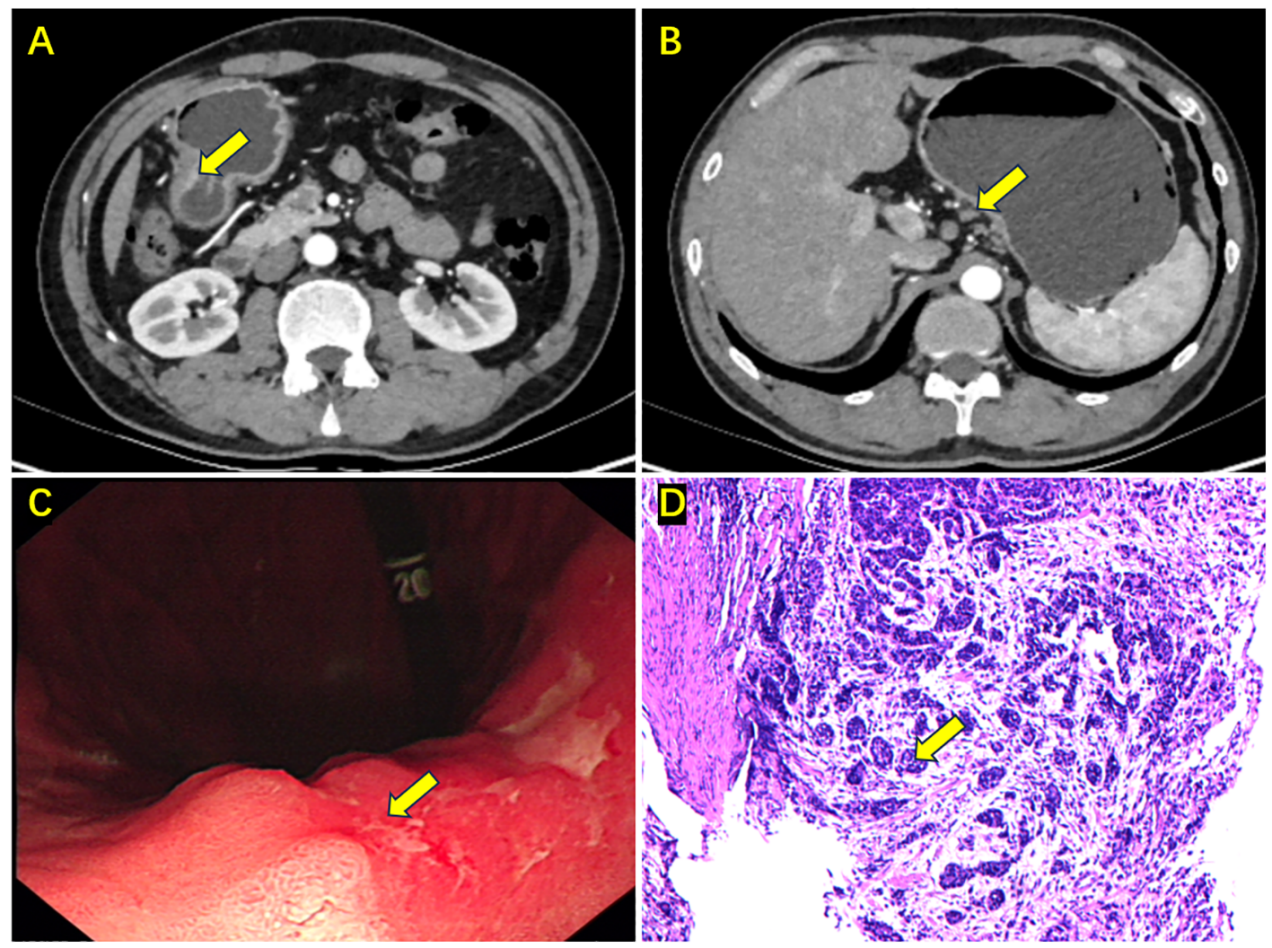
Enhanced abdominal CT, gastroscopy, and biopsy results upon initial admission. **(A)** Cancer in the gastric antrum (the yellow arrow); **(B)** Enlarged lymph node on the lesser curvature side (the yellow arrow); **(C)** Gastroscopy showing a mass in the gastric antrum (the yellow arrow); **(D)** Biopsy pathology indicating moderately differentiated adenocarcinoma (the yellow arrow).

**Figure 2:**
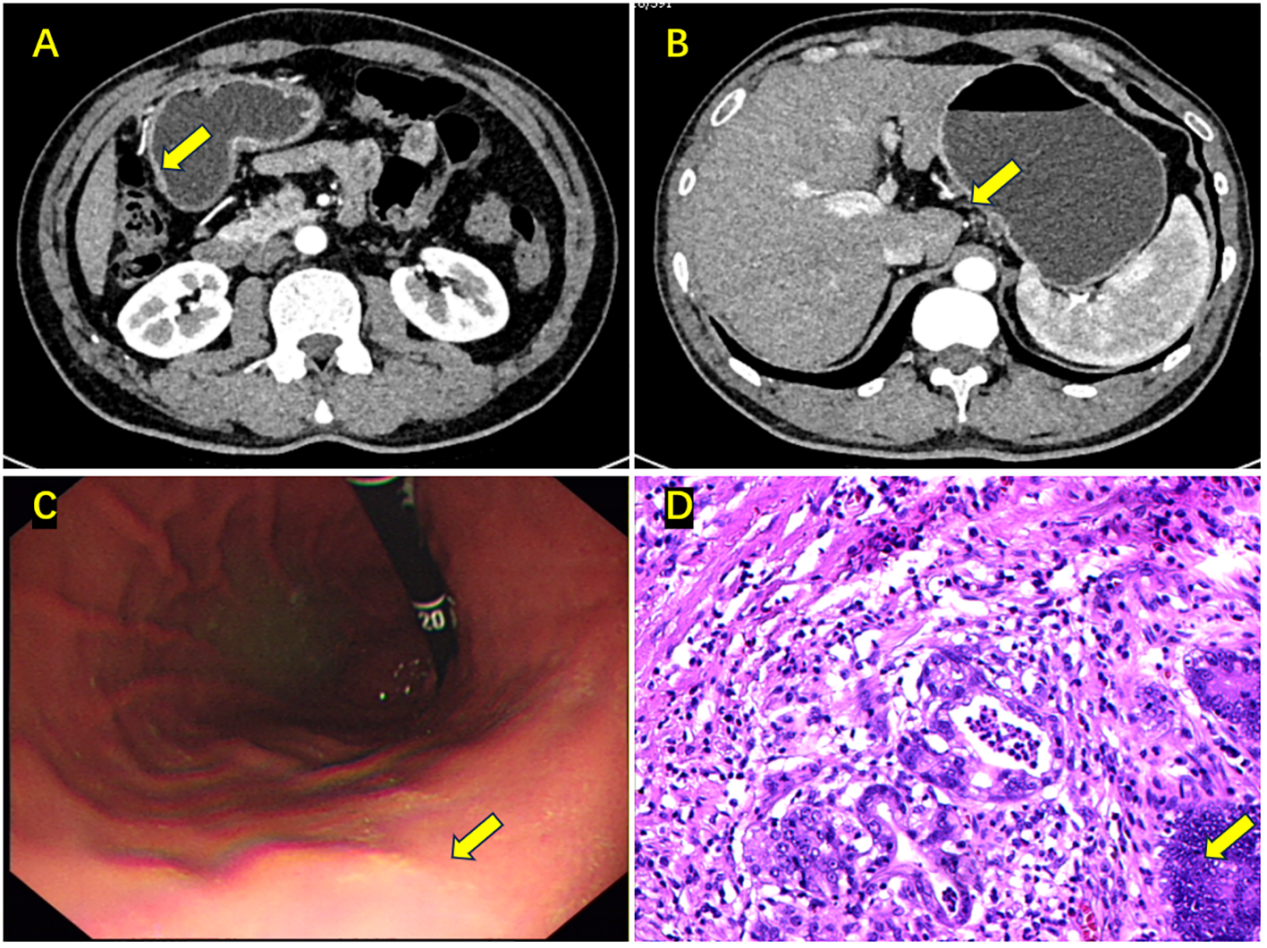
Enhanced abdominal CT, gastroscopy, and postoperative pathology results after neoadjuvant chemotherapy. **(A)** The tumor in the gastric antrum has almost disappeared, with a rough gastric wall (the yellow arrow); **(B)** The enlarged lymph node on the lesser curvature side has disappeared (the yellow arrow); **(C)** The mass in the gastric antrum has disappeared, with mucosal congestion and edema (the yellow arrow); **(D)** Postoperative pathology: ypT1aN0M0, tumor nests located in the lamina propria (the yellow arrow).

A 52-year-old male patient presented to a local hospital with intermittent upper abdominal pain persisting for one year, which worsened over the past month with melena. Gastroscopy and biopsy performed on December 20, 2020, revealed a gastric antral ulcer, adenocarcinoma, and chronic atrophic gastritis. | Gastroscopy and biopsy performed on December 20, 2020, revealed a gastric antral ulcer, adenocarcinoma, and chronic atrophic gastritis. For further evaluation and management, the patient was admitted to the Department of Surgical Oncology at the Second Hospital of Lanzhou University. Abdominal examination was unremarkable. Patient anthropometry: height 170 cm, weight 55 kg, BMI 19.0 kg/m², ECOG PS 1. Gastroscopy showed a 2 cm × 3 cm protruding lesion in the gastric antrum with a central ulcer and indistinct borders. Pathological examination of three biopsy samples confirmed adenocarcinoma in the gastric antrum. Contrast-enhanced abdominal CT (**Figures 1A, B**) demonstrated slight thickening of the gastric angle wall, consistent with cT3N1M0 gastric antral cancer. PET-CT (**Supplementary Figure S1**) scan did not find the matastasis. Following a multidisciplinary team (MDT) discussion, the patient was diagnosed with moderately differentiated adenocarcinoma of the antrum (**Figures 1C, D**), cT3N1M0. The treatment regimen included camrelizumab 200 mg on Day 1 every 3 weeks; apatinib mesylate 0.25 g daily from Day 1 to 21 every 3 weeks; S-1 60 mg twice daily from Day 1 to 14 every 3 weeks; and oxaliplatin 225 mg on Day 1 every 3 weeks. After three cycles of this regimen, contrast-enhanced abdominal CT (**Figures 2A, B**) revealed near-complete resolution of the tumor, with thickening of the gastric antrum wall and no evident enlarged lymph nodes. Gastroscopy (**Figure 2C**) showed resolution of the antral mass with hyperemic and edematous mucosa.

On April 16, 2021, the patient underwent laparoscopic distal subtotal gastrectomy with D2 lymph node dissection. The postoperative pathology report (**Figure 2D**) indicated that most of the stomach angle mucosa showed chronic inflammation with numerous lymphocytes and a few foam cells infiltrating. Immunohistochemical staining revealed focal residual cancer cells (Lauren classification: intestinal type), tumor regression grade (Becker classification: Grade 1b), with the tumor located in the lamina propria (pT1a). No definite invasion of nerves or vessels was observed, and no residual cancer was found at either resection margin. Lesser curvature lymph nodes: 0/5, no cancer metastasis. Greater curvature lymph nodes: 0/2, no cancer metastasis. Additional lymph nodes: Total (0/38), no cancer metastasis. Specific groups: 2 (0/1), 3 (0/1), 4 (0/5), 5 (0/5), 6 (0/3), 7 (0/13), 8 (0/6), 10 (0/3), 11p (0/1). Immunohistochemical staining revealed scattered residual cancer cells positive for CK8/18 and p53 (wild-type). C-erbB-2 (1+), PMS-2 (+), MLH-1 (+), MSH-6 (+), MSH-2 (+), 4% PD-L1 positive cells, CPS=6, and 40% Ki67-positive cells; histiocytes were CD68 positive. For immunohistochemistry, specific monoclonal antibodies (product number) purchased from Fuzhou New Step Biotechnology Development Company were used to stain the tissue sections, including [anti-cytokeratin-8/18 (MAB-1002), anti-p53 (MAB-0674), anti-Her-2 (Kit-0043), anti-PMS2 (MAB-0859), anti-MLH-1 (MAB-0789), anti-MSH6 (MAB-0831), anti-MSH2 (MAB-0836), anti-PD-L1 (MAB-0889), anti-Ki67 (MAB-0672), CD68 (Kit-0026)]. Immunohistochemical slides were independently reviewed by three experienced pathologists. The evaluation of protein expression combined their assessments with quantitative scoring using ImageJ software. Discrepancies in interpretation were resolved through consensus meetings. The patient had a good postoperative recovery and was subsequently discharged.

**Figure 3:**
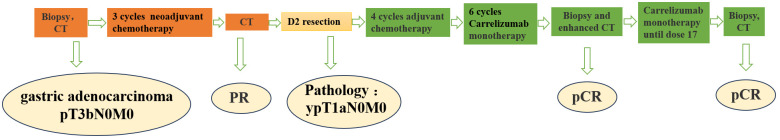
Treatment flow chart (CT, Computerized Tomography, PR, Partial Response, D2, Dissection 2 Lymphadenectomy, pCR, Pathological Complete Response).

**Figure 4:**
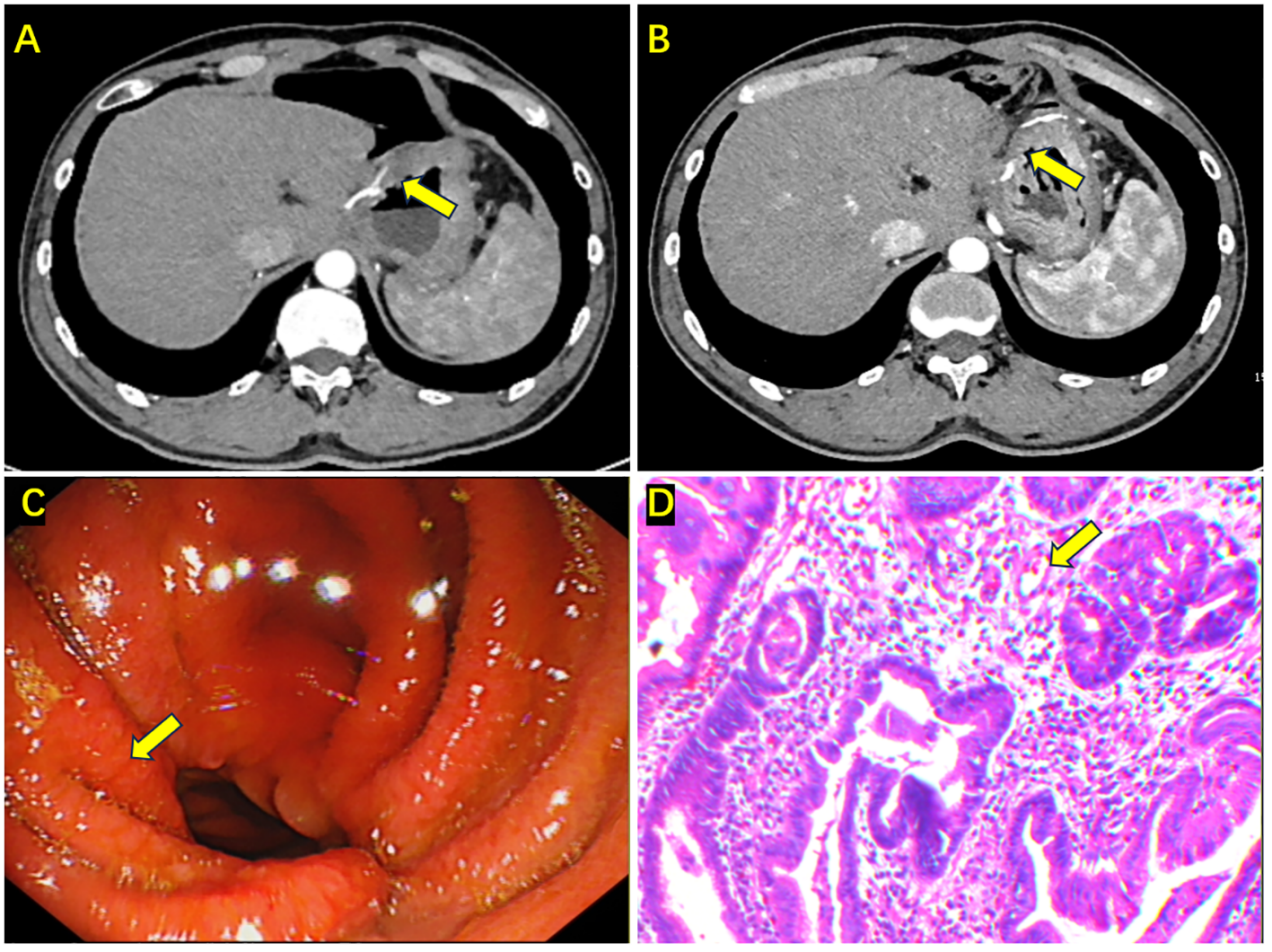
Enhanced abdominal CT, gastroscopy, and biopsy results after completing all treatments. **(A, B)** Enhanced CT shows edema and thickening at the anastomosis, with no obvious enhancement (the yellow arrow); **(C)** Gastroscopy showing congestion and edema at the anastomosis, with a rough mucosa (the yellow arrow); **(D)** Biopsy indicating chronic mucosal inflammation, epithelial hyperplasia, interstitial edema, and no apparent tumor cells in the tissue (the yellow arrow).

After a four-week rest post-surgery, the patient received four cycles of treatment with tigatuzumab, oxaliplatin, apatinib mesylate, and camrelizumab from May 18, 2021, to August 14, 2021. From September 15, 2021, to September 26, 2023, the patient underwent maintenance therapy with camrelizumab, totaling 17 doses (including preoperative and postoperative, **Figure 3**). Following the completion of treatment, enhanced abdominal CT indicated a good postoperative condition of the distal stomach, with no significant thickening or abnormal enhancement at the anastomosis, and no evident distant metastasis (**Figures 4A, B**). Gastroscopy and biopsy revealed mucosal edema and thickening at the anastomosis, with a rough surface (**Figure 4C**). The biopsy pathology showed chronic mucosal inflammation, epithelial hyperplasia, and interstitial edema, with no apparent tumor cells (**Figure 4D**). The therapeutic efficacy was evaluated as ypT0N0M0, achieving pathological complete response (pCR). The last follow-up was on May 29, 2024. The ECOG PS was 1, weight was 61 kg, and BMI was 21.1 kg/m². The patient occasionally experienced dyspepsia. The results of gastroscopy and enhanced abdominal CT are shown in **Supplementary Figure S2**. The patient experienced side effects in the process including Dyspepsia (CTCAE Grade 1), nausea (CTCAE Grade 2), vomiting (CTCAE Grade 3), fatigue (CTCAE Grade 2), epistaxis (CTCAE Grade 1), oropharyngeal pain (CTCAE Grade 2), numbness of the upper limbs (CTCAE Grade 1), surgical wound dehiscence with bleeding (CTCAE Grade 1), increased thyroid-stimulating hormone (CTCAE Grade 1), decreased thyroxine (CTCAE Grade 1), capillary proliferation (CTCAE Grade 1), bone marrow suppression reactions including leukopenia (CTCAE Grade 1) and thrombocytopenia (CTCAE Grade 2).

## Discussion

The recurrence rate of locally advanced gastric cancer treated solely with surgery is high. In Western countries, the recurrence rate within five years is approximately 65–75%, while in Japan, it is less than 50% (7, 8). Based on the MAGIC study (7) and the FNCLCC/FFCD (9) study, perioperative chemotherapy has become the standard treatment for locally advanced gastric cancer in Europe. The 2021 CSCO guidelines recommend that neoadjuvant therapy before surgery can improve R0 resection and pathological remission rates, thereby leading to survival benefits. Therefore, for patients with preoperative stage cIII, a comprehensive treatment model of “neoadjuvant therapy + surgery + adjuvant therapy” can be adopted. However, compared to surgery alone, perioperative chemotherapy plus surgery in patients with high levels of microsatellite instability (MSI-H) is associated with poorer overall survival rates (10). The CLASSIC study found that in Asian patients with locally advanced MSI-H gastric cancer, adjuvant therapy with capecitabine and oxaliplatin did not show significant survival benefits (5-year disease-free survival rate of 83.5% vs. 85.7%, p = 0.931) (11, 12). A meta-analysis of four clinical trials (MAGIC, CLASSIC, ARTIST, ITACA-S) showed that, compared to the surgery-alone group, only non-MSI-H patients with locally advanced gastric cancer benefited from chemotherapy plus surgery, with 5-year overall survival rates of 62% and 53%, respectively (HR = 0.75, 95% CI 0.60 ~ 0.94) (13). Therefore, for some cases of locally advanced gastric cancer, relying solely on perioperative chemotherapy may not achieve the expected therapeutic effect. Some studies have shown that combining immune checkpoint inhibitors with chemotherapy can enhance the efficacy in treating various cancers, such as non-small cell lung cancer and renal cell carcinoma (14, 15). This may be due to chemotherapy activating endogenous anti-tumor immune responses, leading to increased expression of co-stimulatory molecules CD80 and CD86 and downregulation of PD-L1, inducing immunogenic tumor cell death (16). Cytotoxic drugs cause tumor cell death and release a large amount of antigens, stimulating the activation of the immune system. Some chemotherapeutic drugs, such as cyclophosphamide, can inhibit Treg cells, reducing their immunosuppressive effects. Additionally, certain chemotherapy drugs can suppress myeloid-derived suppressor cells (MDSCs) and IFN-γ (17).

A meta-analysis incorporating five randomized controlled clinical trials (18) with 3,355 patients demonstrated that, compared to chemotherapy alone, combined immunotherapy yielded a higher objective response rate (OR = 0.63, 95% CI: 0.55–0.72, P<0.00001), longer overall survival (OR = 0.63, 95% CI: 0.55–0.72, P<0.00001), and progression-free survival (HR=0.75, 95% CI: 0.69–0.82, P<0.00001). Therefore, for the first-line treatment of advanced gastroesophageal cancer, the efficacy of ICI combined with chemotherapy surpasses that of chemotherapy alone. Another meta-analysis (19), which included four randomized controlled clinical trials with 2,253 patients with gastric or gastroesophageal junction cancer, indicated that PD-1/PD-L1 inhibitors significantly extended overall survival (HR = 0.91, 95% CI: 0.83–1.00, P=0.04), although there was no significant change in progression-free survival (HR = 0.91, 95% CI: 0.83–1.00, P=0.04). For other types of cancer, we only found a case report (20) of a patient diagnosed with esophageal neuroendocrine carcinoma after esophagectomy who used two cycles of first-line paclitaxel liposome and S-1 and second-line apatinib and S-1 for two months, but both treatments resulted in progressive disease. Finally, the patient received salvage camrelizumab plus apatinib for relapse and demonstrated a progression-free status for more than ten months following the combination therapy.

Apatinib mesylate is a small molecule VEGFR-2 tyrosine kinase inhibitor and was the first small molecule targeted drug proven to improve the survival rate of patients with gastric cancer, hepatocellular carcinoma, and non-small-cell lung cancer (21). In 2014, apatinib mesylate received approval from the China National Medical Products Administration for the treatment of advanced gastric cancer in the third line and beyond, providing a new treatment option for patients who failed second-line therapy. Therefore, it is worth further exploring whether the combination of perioperative chemotherapy, immunotherapy, and small molecule targeted therapy can achieve better treatment outcomes.

A 2021 clinical trial (22) showed that 48 patients with advanced gastric or gastroesophageal junction adenocarcinoma who did not progress after 4 to 6 cycles of treatment with camrelizumab combined with CAPOX subsequently received treatment with camrelizumab combined with apatinib mesylate. The objective response rate of this combination therapy was 58.3%. The median duration of response was 5.7 months, the median overall survival was 14.9 months, and the median progression-free survival was 6.8 months. Another study (23) evaluated the efficacy of camrelizumab combined with apatinib mesylate and tigatuzumab as second-line treatment for advanced gastric or gastroesophageal junction adenocarcinoma. Among 24 patients, 7 (29.2%) achieved an objective response. The median progression-free survival was 6.5 months. The study demonstrated that regardless of PD-L1 expression, this combination therapy showed promising antitumor activity with controllable toxicity. A clinical trial in 2023 (24) evaluated the efficacy of neoadjuvant/conversion therapy with camrelizumab plus apatinib mesylate and SOX for cT4a/bN+ gastric cancer. The results showed that the complete and major pathological response rates were 15.8% and 26.3%, respectively. Pathological response was significantly associated with microsatellite instability status, PD-L1 expression, and tumor mutation burden. The study concluded that neoadjuvant/conversion therapy based on ICI and anti-angiogenesis showed good efficacy and feasibility in cT4a/bN+ gastric cancer, especially in patients with MSI-H and PD-L1 positivity.

As mentioned, preliminary results of studies (3–6) of camrelizumab plus apatinib mesylate and SOX indicated a good R0 resection rate, ORR rate, pCR rate, and an encouraging median event-free survival of 42.6 months. This patient achieved R0 resection, pCR, has survived 40 months, and still has a good performance status. In this case, the expression of mismatch repair genes (PMS-2, MLH-1, MSH-2, MSH-6) was positive, indicating MSI-L or MSS status. The proportion of PD-L1 positive cells is 4%, and CPS is 6. Tumor mutation burden data was not detected due to financial constraints. According to KEYNOTE-059 (25), patients with PD-L1-positive versus PD-L1-negative gastric cancer had a significantly improved objective response rate (15.5% vs. 6.4%) and median duration of response (16.3 vs. 6.9 months). This demonstrates that the patient truly benefited from camrelizumab.

In summary, neoadjuvant therapy improves R0 resection and pathological response rates, thereby enhancing survival. The key to successful comprehensive treatment of gastric cancer lies in selecting effective and tolerable treatment regimens. Preliminary studies provide a basis for the feasibility and safety of combining perioperative chemotherapy with VEGFR inhibitors and immunotherapy in locally advanced gastric cancer.

## Data availability statement

The original contributions presented in the study are included in the article/**Supplementary Material**. Further inquiries can be directed to the corresponding author.

## Ethics statement

The studies involving humans were approved by Ethics Committee of the Second Hospital of Lanzhou University. The studies were conducted in accordance with the local legislation and institutional requirements. The participants provided their written informed consent to participate in this study. Written informed consent was obtained from the individual(s) for the publication of any potentially identifiable images or data included in this article.

## Author contributions

JH: Data curation, Writing – original draft. XL: Formal analysis, Writing – review & editing. YW: Resources, Writing – review & editing. BX: Project administration, Writing – review & editing. PH: Data curation, Writing – review & editing. ZW: Investigation, Writing – review & editing. LH: Investigation, Writing – review & editing. HC: Writing – review & editing.

## Supplementary material

Supplementary Figure 1 Pet-CT shows there is no metastasis of cancer.

Supplementary Figure 2 Enhanced abdominal CT, gastroscopy, and biopsy results in May 29, 2024. A and B. Enhanced CT shows edema and thickening at the anastomosis, with no obvious enhancement (the yellow arrow); C. Gastroscopy shows congestion and edema at the anastomosis, with a rough mucosa (the yellow arrow); D. Biopsy indicates chronic mucosal inflammation, interstitial edema, and no apparent tumor cells in the tissue (the yellow arrow).
