# Supplementary figures and images for: SOX combined with apatinib and camrelizumab in the treatment of resectable locally advanced gastric cancer: a case report
Source: Front Immunol. 2024 Jul 12;15:1410284. doi: 10.3389/fimmu.2024.1410284 (PMC11272450; doi:10.3389/fimmu.2024.1410284)

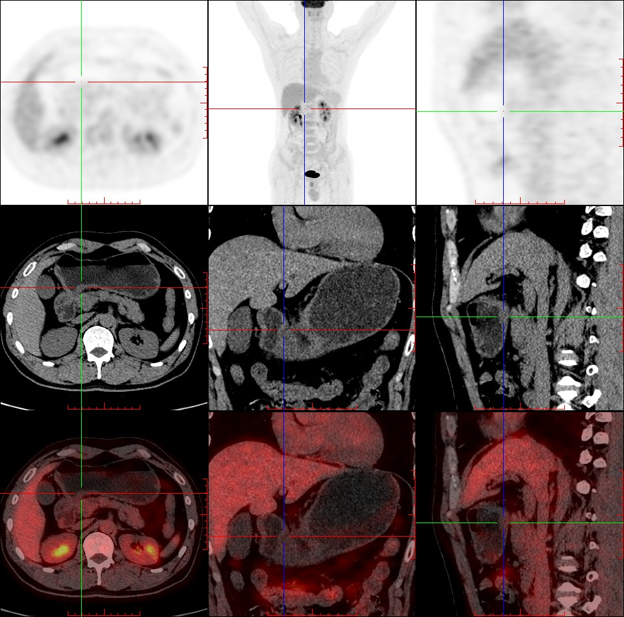

Supplement: Supplementary Figure 1 — Pet-CT shows there is no metastasis of cancer. [file Image_1.tif]

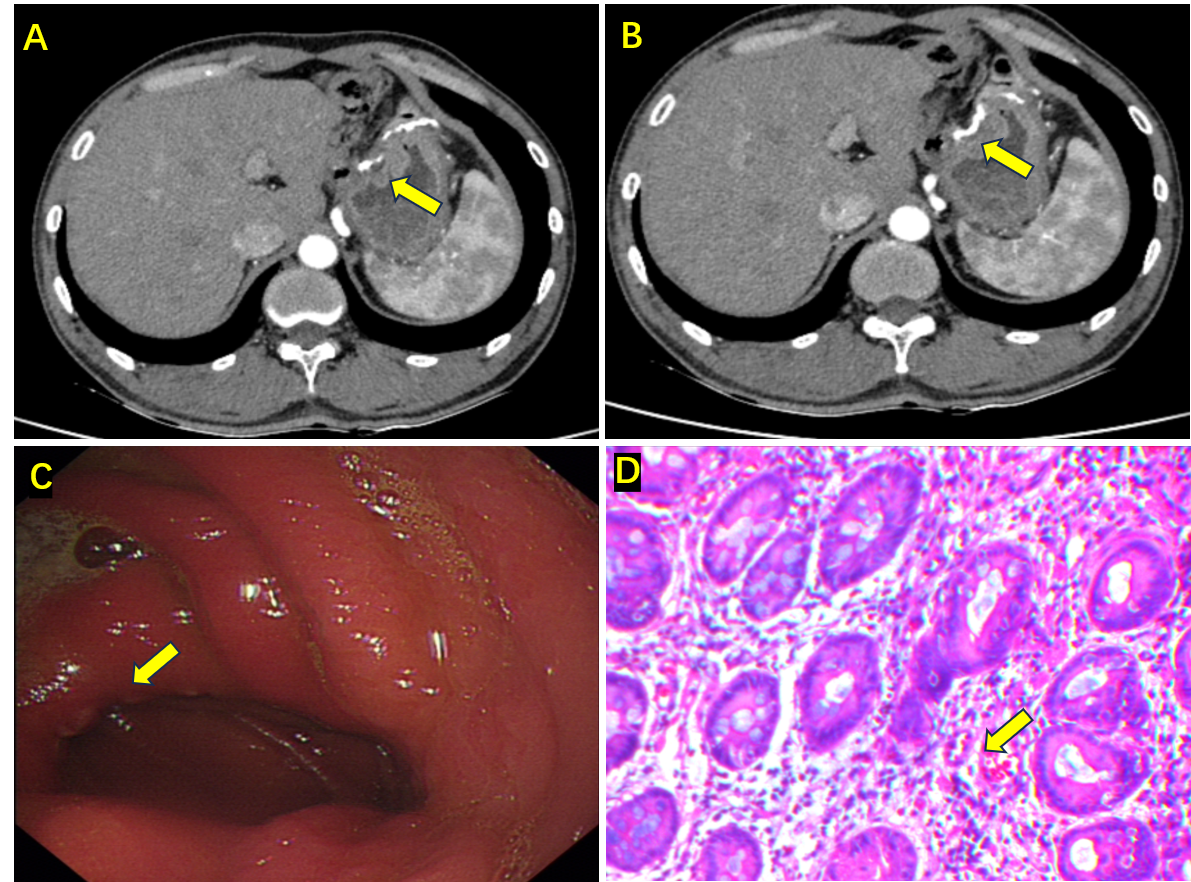

Supplement: Supplementary Figure 2 — Enhanced abdominal CT, gastroscopy, and biopsy results in May 29, 2024. A and B. Enhanced CT shows edema and thickening at the anastomosis, with no obvious enhancement (the yellow arrow); C. Gastroscopy shows congestion and edema at the anastomosis, with a rough mucosa (the yellow arrow); D. Biopsy indicates chronic mucosal inflammation, interstitial edema, and no apparent tumor cells in the tissue (the yellow arrow). [file Image_2.tif]
